# Metabolism and Biodegradation of Spacecraft Cleaning Reagents by Strains of Spacecraft-Associated *Acinetobacter*

**DOI:** 10.1089/ast.2017.1814

**Published:** 2018-11-29

**Authors:** Rakesh Mogul, Gregory A. Barding, Sidharth Lalla, Sooji Lee, Steve Madrid, Ryan Baki, Mahjabeen Ahmed, Hania Brasali, Ivonne Cepeda, Trevor Gornick, Shawn Gunadi, Nicole Hearn, Chirag Jain, Eun Jin Kim, Thi Nguyen, Vinh Bao Nguyen, Alex Oei, Nicole Perkins, Joseph Rodriguez, Veronica Rodriguez, Gautam Savla, Megan Schmitz, Nicholas Tedjakesuma, Jillian Walker

**Affiliations:** Chemistry and Biochemistry Department, California State Polytechnic University, Pomona (Cal Poly Pomona), Pomona, California.

**Keywords:** Acinetobacter, Planetary protection, Extreme survival, Metabolism, Spacecraft, Cleaning, Bioburden

## Abstract

Spacecraft assembly facilities are oligotrophic and low-humidity environments, which are routinely cleaned using alcohol wipes for benchtops and spacecraft materials, and alkaline detergents for floors. Despite these cleaning protocols, spacecraft assembly facilities possess a persistent, diverse, dynamic, and low abundant core microbiome, where the *Acinetobacter* are among the dominant members of the community. In this report, we show that several spacecraft-associated *Acinetobacter* metabolize or biodegrade the spacecraft cleaning reagents of ethanol (ethyl alcohol), 2-propanol (isopropyl alcohol), and Kleenol 30 (floor detergent) under ultraminimal conditions. Using cultivation and stable isotope labeling studies, we show that ethanol is a sole carbon source when cultivating in 0.2 × M9 minimal medium containing 26 μM Fe(NH_4_)_2_(SO_4_)_2_. Although cultures expectedly did not grow solely on 2-propanol, cultivations on mixtures of ethanol and 2-propanol exhibited enhanced plate counts at mole ratios of ≤0.50. In support, enzymology experiments on cellular extracts were consistent with oxidation of ethanol and 2-propanol by a membrane-bound alcohol dehydrogenase. In the presence of Kleenol 30, untargeted metabolite profiling on ultraminimal cultures of *Acinetobacter radioresistens* 50v1 indicated (1) biodegradation of Kleenol 30 into products including ethylene glycols, (2) the potential metabolism of decanoate (formed during incubation of Kleenol 30 in 0.2 × M9), and (3) decreases in the abundances of several hydroxy- and ketoacids in the extracellular metabolome. In ultraminimal medium (when using ethanol as a sole carbon source), *A. radioresistens* 50v1 also exhibits a remarkable survival against hydrogen peroxide (∼1.5-log loss, ∼10^8^ colony forming units (cfu)/mL, 10 mM H_2_O_2_), indicating a considerable tolerance toward oxidative stress under nutrient-restricted conditions. Together, these results suggest that the spacecraft cleaning reagents may (1) serve as nutrient sources under oligotrophic conditions and (2) sustain extremotolerances against the oxidative stresses associated with low-humidity environments. In perspective, this study provides a plausible biochemical rationale to the observed microbial ecology dynamics of spacecraft-associated environments.

## 1. Introduction

Spacecraft assembly, test, launch, and operational procedures that minimize the biological contamination of explored environments are critical to ensuring the integrity of future life-detection missions, and in mitigating irreversible impacts to any native biochemical states (Space Studies Board, 2000, 2006). To assist in reducing the probability of contamination, NASA planetary protection requirements for Mars include the assembly of spacecraft in clean room facilities, trajectory biasing for outgoing spacecraft, low impact probabilities for orbiting spacecraft, and partial sterilization of lander and rover spacecraft (at the sub- or full-system levels) (NASA, 2011; Frick *et al.*, 2014).

For spacecraft assembly, all Mars-bound spacecraft (orbiters, landers, and rovers) are additionally subject to requirements necessitating clean rooms with high particulate control (ISO class 8, Class 100,000 or better), proper garmenting procedures for human personnel in the clean rooms (*e.g.*, bunny suit coveralls), and routine cleaning procedures for spacecraft, surfaces, and floors within the assembly facilities (NASA, 2011; Frick *et al.*, 2014). The commonly used cleaning reagents for these purposes are ethanol (ethyl alcohol) and 2-propanol (isopropyl alcohol, isopropanol) for benchtops and spacecraft materials (Barengoltz, 1997; Benardini *et al.*, 2014; Frick *et al.*, 2014), and Kleenol 30 for the clean room floors (Vaishampayan *et al.*, 2013; Benardini *et al.*, 2014; Mahnert *et al.*, 2015). However, despite these practices, spacecraft assembly facilities possess a persistent, yet low abundant core microbiome (∼10^1^–10^2^ colony forming units (cfu)/cm^2^, ∼0.2–300 spores/m^2^, ∼1–40 OTU/m^2^), with molecular genetics revealing a taxonomically diverse and dynamic microbial community (Venkateswaran *et al.*, 2001; Moissl *et al.*, 2007; Vaishampayan *et al.*, 2010; La Duc *et al.*, 2012).

Among the more diverse members of this core microbiome are the *Acinetobacter*, a Gram-negative, nonspore forming, and strictly aerobic genus commonly found in soil and water environments, and increasingly associated with multiantibiotic resistance outbreaks in varying clinical settings (Bergogne-Bérézin *et al.*, 2008). In the context of spacecraft microbiology, nonpathogenic strains of *Acinetobacter* have been isolated and detected in diverse spacecraft-associated environments, including the surface of the preflight Mars Odyssey orbiter (La Duc *et al.*, 2003), floors in the assembly facility for the Mars Phoenix lander (Ghosh *et al.*, 2010), surfaces in the assembly facilities for the Herschel spacecraft (Moissl-Eichinger *et al.*, 2013), surfaces of the International Space Station (ISS) (Castro *et al.*, 2004), and in the drinking water of the ISS (La Duc *et al.*, 2004). Molecular community analyses further suggest that the diversity (and/or abundance) of *Acinetobacter* increases during spacecraft assembly, as was observed for the Mars Phoenix lander, where the relative abundance of *Acinetobacter* among all genera (in operational taxonomic units) increased ∼10-fold upon commencement of assembly and enforcement of the surface and floor cleaning protocols (Vaishampayan *et al.*, 2010).

In fact, for the Mars Phoenix lander, phylogenetic changes across several genera were quantitated over the course of the spacecraft assembly process, which included sampling before, during, and after assembly (Vaishampayan *et al.*, 2010). Together, these net phylogenetic changes (before vs. after assembly) showed (1) relative increases in abundance for the *Acinetobacter*, *Ralstonia*, and *Brevundimonas* (all Gram-negative), (2) relative decreases for *Mycoplana*, *Sphingomonas*, and *Pseudomonas*, and (3) <1% or no net changes for most Gram-positive genera such as *Streptococcus* and the spore-forming *Bacillus*.

In further detail, and as a comparative example, the *Acinetobacter* and *Streptococcus*, respectively, increased ∼10 and ∼100-fold during the assembly process (from ∼4% to ∼38% and from ∼0.4% to ∼55%, respectively), likely due to contamination arising from increased personnel and activities within the facilities. However, after assembly, and despite the routine cleaning procedures, the *Acinetobacter* further increased to ∼48% (or a net ∼10-fold increase) to ultimately represent the numerically dominant genus within the postassembly community. In contrast, the *Streptococcus* reduced ∼180-fold in abundance and reduced to 0.3%.

These observations of a dynamic and persistent spacecraft microbial community support the hypothesis that the core microbiome is composed of members that harbor a biochemical potential to tolerate the cleaning procedures, and survive the oligotrophic and low-humidity environments of the assembly facilities (La Duc *et al.*, 2007, 2012; Moissl-Eichinger *et al.*, 2013). Accordingly, the objective of this work was to measure the ability of spacecraft-associated *Acinetobacter* to metabolize and biodegrade spacecraft cleaning reagents, and survive under extreme conditions, when cultivated under nutrient-restricted conditions.

## 2. Materials and Methods

### 2.1. Materials

Spacecraft-associated *Acinetobacter* strains were obtained from the Planetary Protection Culture Collection at the Jet Propulsion Laboratory (Pasadena, CA) and included *Acinetobacter radioresistens* 50v1, *Acinetobacter proteolyticus* 2P01AA (formerly assigned as *Acinetobacter gyllenbergii* 2P01AA), *Acinetobacter johnsonii* 2P08AA, *A. johnsonii* 2P07AA, *Acinetobacter oryzare* 2P08MC, *Acinetobacter guillouiae* 2P07PB, and *A. guillouiae* 2P07PC. The control type strain, *A. radioresistens* 43998^T^, was obtained from the American Type Culture Collection.

The spacecraft cleaning reagents of ethanol (Omnipur Pure, 200 proof; VWR), 2-propanol (Fisher Sci.), and Kleenol 30 (Mission Laboratories, Los Angeles, CA; Clovis Janitorial) were sterile filtered, without dilution, and saved as aliquots at 4°C. Concentrated 5 × minimal medium (M9) was prepared using 64.0 g Na_2_HPO_4_·7H_2_O (Amresco), 15.0 g KH_2_PO_4_ (EM Science), 2.5 g NaCl (EM Science), and 5.0 g NH_4_Cl (EM Science) per liter water. To a 200 mL aliquot of 5 × M9 medium, 2.0 mL of 1 M MgSO_4_ (EM Science) and 100 μL 1 M CaCl_2_ (EM Science) were added, and the total solution was diluted to 1 L using water to yield 1 × M9; in turn, this medium was further diluted fivefold to yield 0.2 × M9. Lysogeny broth (LB) medium was prepared using 10.0 g tryptone (VWR Amresco), 5.0 g yeast (Becton, Dickinson and Company), 10.0 g NaCl (EM Science), and 1.0 mL of 1 M NaOH (Sigma-Aldrich) per liter of water.

Agar plates were prepared using 1 L LB medium and 15 g of bacteriological agar (AMRESCO). Stock solutions of 10 mM Fe^2+^ were prepared by fully dissolving 0.19607 g of Fe(NH_4_)_2_(SO_4_)_2_·6H_2_O (EM Science) in 50.0 mL water, followed by sterile filtration, and storage as aliquots at 4°C. Buffers included 4-(2-hydroxyethyl)-1-piperazineethanesulfonic acid (HEPES; VWR) and phosphate-buffered saline (PBS, G-Biosciences). Temporal changes in cell density were followed by optical density (OD) measurements at 600 nm (Spectronic 20 Genesys), and by plate counts, which were expressed as cfu/mL. All microbiology media were autoclaved at 121°C for 30 min, buffers and metal solutions were sterile filtered using 0.22 μm cellulose acetate filters (VWR), and ultrapure water (18 MΩ cm^−1^) was used throughout. Solutions of 20 mM nicotinamide adenine dinucleotide (NAD^+^; Sigma-Aldrich) and 10 mM 2,3-bis-(2-methoxy-4-nitro-5-sulfophenyl)-2H-tetrazolium-5-carboxanilide (XTT; Amresco) were prepared in water and sterile filtered, where NAD^+^ was stored as aliquots at −20.0°C and XTT was freshly prepared.

### 2.2. Ultraminimal cultivations with spacecraft cleaning reagents

All cultivations were performed in ultraminimal medium (0.2 × M9) containing 9.5 mM Na_2_HPO_4_, 4.4 mM KH_2_PO_4_, 1.7 mM NaCl, 3.7 mM NH_4_Cl, 0.4 mM MgSO_4_, and 20 μM CaCl_2_. For this study, 0.2 × M9 was supplemented with Fe(NH_4_)_2_(SO_4_)_2_ to provide the sole added transition metal of 26 μM Fe^2+^. Cultivations in this medium were performed using (1) ethanol concentrations ranging from 2 to 650 mM, (2) 200 mM mixtures of ethanol and 2-propanol, using the respective mole ratios of 0, 0.5, 0.85, and 1, and (3) mixtures of 16 mM ethanol (0.1% v/v) containing 0.1% or 1.0% v/v Kleenol 30. Cultivations were also performed using stable isotopes of ethanol, where stationary phase cultures of *A. radioresistens* 50v1 (OD ∼0.9, ∼9 × 10^8^ cfu/mL) were prepared under ultraminimal conditions (0.2 × M9, 26 μM Fe^2+^) using 16 mM ethanol or [1,2-^13^C_2_] ethanol as the sole carbon source.

All preinoculate cultures were prepared in conditions that matched those of the respective final culture (as already listed), and were inoculated using an isolated colony obtained from LB agar plates, which were prepared from streaks of glycerol stocks of the selected isolate. All preinoculate cultures were 2 or 5 mL in volume (using 13 × 100 mm and 10 × 1.5 cm cultures tubes, respectively), agitated at 32°C at 200 rpm, and grown until mid-log phase (OD ∼0.5–0.6, or ∼2 × 10^8^–4 × 10^8^ cfu/mL) or late-log phase (OD ∼0.6–0.7, ∼5 × 10^8^–7 × 10^8^ cfu/mL). Fresh medium was inoculated with 1:100 volume of the preinoculate, grown at 32°C at 200 rpm, and treated as described.

Cultivations were performed using capped and parafilmed culture vessels (threaded screw caps for tubes; sterile rubber plugs or aluminum foil for Erlenmeyer flasks). In comparison, cultivations performed in loosely capped culture vessels yielded reliable growth, but irreproducible phenotypic measures, most likely due to evaporation of ethanol. Control experiments included cultures containing all components of the medium except the bacterial inoculum (inoculum-negative) or ethanol carbon source (ethanol-negative); in all cases, the controls yielded no growth.

### 2.3. Growth rates and plate counts

Growth rates were determined by nonlinear regression (KaleidaGraph, Synergy Software; and Microsoft Excel) using a modified version of the Gompertz equation [Eq. (1)] (Begot *et al.*, 1996). Regression parameters included the time of measurement (*t*), growth rate (*k*), lag time (*L*), and the maximum $\log \left( { { \frac { \Delta OD }  { \Delta O { D_ { { \rm { min } } } } } } } \right)$ value (*A*). For the regressions, the final OD values for the inoculum-negative controls were negligible, thereby indicating no measurable contamination or formation of nonbiological particulate matter after cultivation.

\begin{align*}
\begin{split}\log \left( {{N \over {{N_0}}}} \right) = \log \left( {{{ \Delta OD} \over { \Delta O{D_{{ \rm{min}}}}}}} \right) \\= A \cdot \exp \left( {- \exp \left( {{{k \cdot e} \over A} \cdot \left( {L - t} \right)+1} \right) } \right).\end{split}
 \tag{1}\end{align*}

For all plate count measurements, aliquots were removed from the cultures, decimally diluted in 0.2 × M9 (by 10–100,000-fold), and spread in 20 μL aliquots onto LB agar plates. After incubation for 15–24 h at 32°C, plates bearing clearly isolated colonies, but no more than ∼300, were enumerated from at least three biological replicates, using at least two technical replicates per trial. For this study, LB agar plates were used for reproducibility purposes, as plating onto M9/ethanol agar plates provided unreliable cell counts (due to inconsistent adsorption of ethanol onto the agar plates across trials, and loss of ethanol and cracking of the agar during incubation). Negligible colony counts were obtained from the inoculum-negative and ethanol-negative controls.

### 2.4. Oxidative extremotolerance under ultraminimal conditions

Cultures (40 mL) of *A. radioresistens* 50v1 were prepared in ultraminimal medium (0.2 × M9, 26 μM Fe^2+^), containing 16 mM ethanol (0.1% v/v), and exposed to hydrogen peroxide (H_2_O_2_). As nutrient-rich controls, cultures (40 mL) were also prepared in LB and exposed to H_2_O_2_. All cultures were grown to mid-log phase (∼2 × 10^8^ cfu/mL in 0.2 × M9; ∼2 × 10^9^ cfu/mL in LB), aseptically divided into equal portions, transferred to 250 mL Erlenmeyer flasks, and treated separately as the respective exposed and unexposed samples.

To the exposed samples, final concentrations of 10, 100, and 400 mM (0.034%, 0.34%, and 1.4% w/v) H_2_O_2_ were added (nonstabilized 30% w/w; Sigma-Aldrich), and incubated in parallel for 1 h at 32°C with constant agitation at 200 rpm. Upon completion, 100 μL of the cultures was quenched 1:10 with 0.1 mg/mL bovine liver catalase (to remove any excess H_2_O_2_). The exposed and unexposed cultures were then decimally diluted (10^4^- and 10^5^-fold dilutions for the 0.2 × M9 samples, and 10^6^-fold dilutions for the LB samples), and 100 μL aliquots were spread onto LB agar plates. Plates were incubated and enumerated as described.

### 2.5. Alcohol dehydrogenase kinetics

The kinetics of alcohol dehydrogenase were measured by absorbance spectroscopy (Beckman Coulter DU640). Mid-log phase cultures (80 mL) of *A. radioresistens* 50v1 were prepared in 0.2 × M9 (with 26 μM Fe^2+^) containing 16 mM ethanol. Cells were harvested by centrifugation at 6000 *g* at 4°C for 10 min (Beckman Coulter Allegra™ 21R), the supernatants were discarded, and cell pellets thoroughly washed by resuspending in 20 mL 1× PBS, harvesting, and repeating the procedure another two times; at the penultimate step, samples were aliquoted and the final cell pellets stored at −80°C.

Protein extracts were prepared from 0.1–0.2 g of the cell pellets (wet mass), which were thawed on ice, resuspended in 10 mL 50 mM HEPES buffer (pH 7.5), and ultrasonicated at 30 s intervals (with 30 s intervals on ice) for a total of 2 min of ultrasonication at a power of 5 (Virtis Virsonic Sonicator). The suspensions were then centrifuged at 6000 *g* for 20 min at 4°C, the supernatants and pellets saved, and independently analyzed for alcohol dehydrogenase activity. Pelleted samples were resuspended in 500 μL of 50 mM HEPES (pH 7.5) containing 1% Triton X-100 (v/v). All samples were stored on ice and immediately analyzed.

As determined in control experiments, reproducible rates (*n* ≥ 3) were only obtained when simultaneously using NAD^+^ and an exogenous electron acceptor, such as XTT or DCIP (2,6-dichloroindophenol), with XTT providing lower overall standard deviations. All downstream kinetic studies were performed using 2.5 mM NAD^+^ and 5.0 mM XTT in 50 mM HEPES buffer (pH 7.5), and substrate concentrations of 0.25–10 mM for ethanol or 2-propanol. Reactions were initiated by the addition of 100–200 μL sample and followed by monitoring the change in absorbance at 470 nm every 2 s for 200–800 s. All reactions were thoroughly but gently mixed, 1 mL in final volume, and performed in 1.0 mL Plastibrand^®^ disposable UV cuvettes at 22°C. Reaction rates (*n* ≥ 3) were determined by linear regression over a minimum of 200 s using an *R*^2^ of ≥0.95, and converted to relevant units using the molar extinction coefficient for XTT (3.70 × 10^4^ M^−1^ cm^−1^). For control purposes, rates of background reduction of XTT by the cellular extracts, in the absence of substrate, were also measured.

Specific activities were expressed as pkat/mg protein (1 pkat = 1 × 10^−9^ katals = conversion of 1 pmole of substrate per second), where protein concentrations of the cell lysates were measured using Bio-Rad standard and DC protein assays following the manufacturer's instructions. Michaelis–Menten pseudoparameters were calculated through nonlinear least-squares fitting of the rate data (IC_50_ Toolkit; ic50.tk), which provided the parameters of maximum specific activity of catalysis (pkat/mg) and *apparent K*_M_ (mM); fits were obtained using aggregated data from several trials (*n* = 7–9, ethanol; *n* = 3–7, 2-propanol), and standard deviation was calculated from the standard error of the regression.

### 2.6. Stable isotope labeling and metabolite profiling

Stationary phase cultures (2 mL) of *A. radioresistens* 50v1 (OD ∼0.9, ∼9 × 10^8^ cfu/mL) were prepared as described using ethanol (natural abundance) or ^13^C_1,2_-labeled ethanol under ultraminimal conditions (0.2 × M9, 26 μM Fe^2+^). All cultures were harvested by centrifugation at 3500 *g* for 15 min at 4°C (Beckman Coulter Allegra 21R), the supernatants were discarded, and resulting cell pellets were washed by resuspending in 2–6 mL 1× PBS. The suspensions were centrifuged again, the supernatant was discarded, and washing procedure was repeated once more. The final cell pellets were partly dried by centrifugal evaporation (∼12 h; CentiVap Console), stored at −80°C, and ultimately analyzed by untargeted analyses of the primary metabolites by gas chromatography–mass spectrometry (GC-MS).

Cell pellets were extracted by thawing the samples on ice, resuspending in a 50/50 mixture of acetonitrile and water (∼1.5 mL/cell pellet), and vortexing for 3–5 min at a setting of 3000 (Vortex Genie Vortexer 2). Next, samples were clarified by centrifugation (12,000 *g*, 4 min, 4°C), and the supernatants carefully transferred to 2 mL centrifuge tubes and immediately dried to ∼10 μL by centrifugal evaporation (≥8 h at medium heat; DNA 110 Savant DNA SpeedVac). The concentrated extracts were diluted ∼100-fold using 1 mL of 50/50 acetonitrile/H_2_O, vortexed again, and transferred in 10 μL aliquots to microvolume glass inserts (American Chromatography), which had been inserted into 2 mL centrifuge tubes. Samples were again dried by centrifugal evaporation (∼10 min, medium heat) and stored at −80°C or immediately prepared for GC-MS analysis.

Samples were derivatized by addition of 10 μL of 20 mg/mL methoxyamine hydrochloride in pyridine (Sigma-Aldrich), followed by incubation at 37°C for 90 min. Samples were then equilibrated to room temperature, mixed with 2 μL of a standard mixture of fatty acid methyl esters (FAMEs, C8–C10, even chains from C12–C30) (Barding *et al.*, 2013), and silylated by addition of 90 μL of *N*-methyl-*N*-(trimethylsilyl)trifluoroacetamide (MSTFA; Sigma-Aldrich) containing 1% trimethylchlorosilane (Sigma-Aldrich), followed by heating at 37°C for 30 min.

Untargeted analyses were also performed on the extracellular fractions of cultures grown in the absence and presence of 0.1% and 1.0% (v/v) Kleenol 30. For these samples, late-log cultures (2 mL) were separated by centrifugation (as described), and the supernatants were removed and separated into 500 μL aliquots, dried by centrifugal evaporation, and stored at −80°C. Upon analysis, the dried samples were directly derivatized using 90 μL MSTFA with 1% trimethylchlorosilane (with FAMEs standards), followed by heating at 37°C for 30 min. All samples were transferred to wide mouth crimp top vials (American Chromatography Supplies, New Jersey) and sealed with an 11 mm crimp cap. Samples were analyzed by GC-MS within 24 h of derivatization.

All samples were analyzed on an Agilent Technologies 6890N Network GC System connected to an Agilent Technologies 5973 Inert Mass Selective Detector and outfitted with a 7683B Series Injector. Separations were performed using an Agilent J&WDB-5ms GC-capillary column (30 m × 0.25 mm i.d.) and an integrated 10 m guard column. Samples (1 μL) were introduced by splitless injection using a constant helium gas (99.999% purity) flow of 1 mL/min. Injection port temperature was maintained at 250°C, and the separation was performed using an initial oven temperature of 60°C (for 1 min) and a temperature ramp of 10°C/min to 320°C (hold for 5 min). Mass spectra were obtained by electron impact ionization at 70 eV, using an ion source temperature of 230°C and collected over a mass range of 60–600 *m/z* (2.71 scans/s).

Deconvolution and identification were performed on the raw data using the Automated Mass Spectral Deconvolution and Identification System software (AMDIS, National Institute of Standards and Technology) using a retention index of ±2% and a corrected match factor of at least 700. The data were integrated using Agilent Mass Hunter Quantitative Analysis B.07.00M after translation with the GC MSD Translator (Agilent). Analyte abundances were compared using parametric *t*-tests, and multiple hypothesis testing was corrected for using a Benjamini–Hochberg false discovery rate of 0.10 (Microsoft Excel).

## 3. Results

### 3.1. Cultivations under ultraminimal conditions

**FIG. 1.**
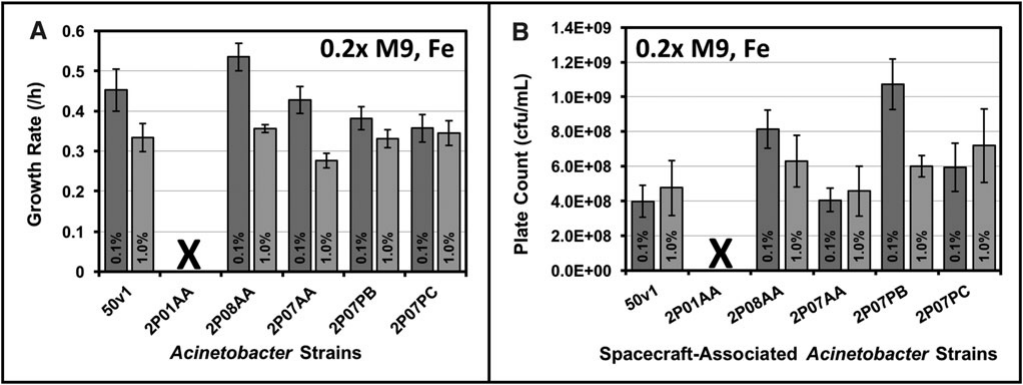
**(A)** Growth rates and **(B)** mid-log phase plate counts for differing strains of spacecraft-associated *Acinetobacter* (50v1, 2P01AA, 2P08AA, 2P07AA, 2P07PB, and 2P07PC) cultivated in 0.2 × M9 (32°C) containing 26 μM Fe^2+^ and 16 mM (0.1% v/v) or 160 mM (1.0% v/v) ethanol; the respective growth curves are provided in Supplementary Figure S1 (X demarks no measurable growth, *n* = 6–7, errors bars in **(A)** represent the standard error of regression and in **(B)** the standard deviation).

Growth rates and viability were measured for multiple spacecraft-associated *Acinetobacter* strains under ultraminimal conditions (0.2 × M9, 16 mM ethanol, 26 μM Fe^2+^). The tested strains included *A. radioresistens* 50v1, which was isolated from the surface of the preflight Mars Odyssey orbiter, and *A. proteolyticus* 2P01AA, *A. johnsonii* 2P08AA, *A. johnsonii* 2P07AA, *A. oryzare* 2P08MC, *A. guillouiae* 2P07PB, and *A. guillouiae* 2P07PC, which were isolated from the floor of the assembly facility for the Mars Phoenix lander. As shown in Figure 1A and Supplementary Fig. S1 (see Supplementary Data at https://www.liebertpub.com/suppl/doi/10.1089/ast.2017.1814), except for the 2P01AA strain, all spacecraft-associated *Acinetobacter* strains grew on ethanol as the sole carbon source, with growth rates ranging from ∼0.36 to 0.53 h^−1^ (in 16 mM or 0.1% v/v ethanol), where the fastest rates were exhibited by *A. johnsonii* 2P08AA (0.53 ± 0.03 h^−1^).

At 10-fold higher concentrations of ethanol (160 mM or 1.0% v/v), rates for the 2P08AA and 2P07AA strains were ∼1.5-fold lower (*p* < 0.05), whereas rates for the 50v1, 2P07PB, and 2P07PC strains were, respectively, and statistically equivalent across 16 and 160 mM ethanol. As displayed in Figure 1B, viable cultures were confirmed by plate counts on mid-log phase cultures, which provided cell densities ranging from ∼4 × 10^8^ to 1 × 10^9^ cfu/mL. Most strains displayed similar cell densities when grown on 16 or 160 mM ethanol; however, plate counts for the 2P07PB strain were ∼1.8-fold higher (1.1 × 10^9^ ± 1.5 × 10^8^ cfu/mL) in 16 mM ethanol.

**FIG. 2.**
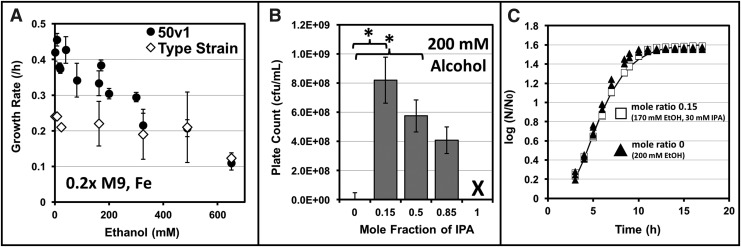
**(A)** Growth rates of *Acinetobacter radioresistens* 50v1 and *A. radioresistens* 43998^T^ cultivated (32°C) in 0.2 × M9 and 26 μM Fe^2+^ containing 2–650 mM ethanol (*n* = 2–6, errors bars represent the standard error of regression); **(B)** mid-log phase plate counts of *A. radioresistens* 50v1 obtained from cultivations (32°C) in 0.2 × M9 and 26 μM Fe^2+^ containing 200 mM ethanol (mole ratio 0), 170 mM ethanol, and 30 mM 2-propanol (mole ratio 0.15), 100 mM ethanol and 100 mM 2-propanol (mole ratio 0.50), 30 mM ethanol and 170 mM 2-propanol (mole ratio 0.85), and 200 mM 2-propanol (mole ratio 1.0) (*demarks statistical significance of *p* < 0.05, X demarks no growth, *n* = 5–11, and error bars represent the standard deviation); and **(C)** growth rates of *A. radioresistens* 50v1 cultivated (32°C) in 0.2 × M9 and 26 μM Fe^2+^ containing 200 mM ethanol (mole ratio 0; triangles) or 170 mM ethanol and 30 mM 2-propanol (mole ratio 0.15; squares).

As a species-level comparison, growth rates were also measured across a range of ethanol concentrations (2–650 mM) for *A. radioresistens* 50v1 and *A. radioresistens* 43998^T^, which served as a nonspacecraft-associated control for this study (Fig. 2A). For the 50v1 strain, rates were fastest at 8 mM ethanol (0.46 ± 0.02 h^−1^) and gradually trended downward between 40 and 650 mM (from 0.43 ± 0.04 to 0.11 ± 0.02 h^−1^). For the type strain, growth rates were essentially equivalent across the range of tested ethanol concentrations; overall, the rates (∼0.22 h^−1^) between ∼2 and 40 mM ethanol were ∼2-fold lower than those of the 50v1 strain. For both strains, growth at concentrations <2 mM provided irreproducible results.

For the 50v1 strain, cultivations were also performed in mixtures of ethanol and 2-propanol using the mole ratios of 0, 0.15, 0.50, 0.85, and 1.0, at a final concentration of 200 mM (Fig. 2B). Using these mixtures, reproducible growth rates and high plate counts (∼10^8^ cfu/mL) were obtained at mole ratios of 0.15, 0.50, and 0.85. For instance, cultures grown in mixtures of 170 mM ethanol and 30 mM 2-propanol (mole fraction of 0.15), 100 mM ethanol and 100 mM 2-propanol (mole fraction of 0.50), and 30 mM ethanol and 170 mM 2-propanol (mole fraction of 0.85) exhibited mid-log phase plate counts of 8.2 × 10^8^ ± 1.6 × 10^8^, 5.8 × 10^8^ ± 1.1 × 10^8^, and 4.1 × 10^8^ ± 0.9 × 10^8^ cfu/mL, respectively.

Comparison of the plate counts (*p* < 0.05) revealed that cell densities obtained at a mole ratio of 0.15 (170/30 ethanol/2-propanol) were ∼2-fold higher than those obtained at 200 mM ethanol (mole ratio of 0; 4.0 × 10^8^ ± 0.5 × 10^8^ cfu/mL), and ∼1.7-fold higher than those obtained (in control experiments) at 170 mM ethanol (4.7 × 10^8^ ± 1.6 × 10^8^ cfu/mL). In contrast, growth rates at the respective mole ratios of 0.15 and 0 (as measured through OD) were statistically equivalent, amounting to 0.33 ± 0.04 and 0.30 ± 0.01 h^−1^ (Fig. 2C). Furthermore, at a mole ratio of 0.50, and when compared with the 200 mM ethanol control, the plate counts were ∼1.5-fold higher (*p* < 0.05), and the growth rate was ∼1.3-fold lower (0.25 ± 0.01 h^−1^). Finally, at a mole ratio of 0.85, and when compared with the 200 mM ethanol control, the plate counts were statistically equivalent, while the growth rate was ∼1.5-fold lower (0.21 ± 0.02 h^−1^).

Together, these trends indicated that cell viabilities at mid-log phase were maximally enhanced in the presence of low mole ratios of 2-propanol, and that a concentration of 30 mM 2-propanol was not inhibitory, as the growth rate was unaffected. At a higher mole ratio of 0.85, however, longer cultivation times were required to reach mid-log phase, as indicated by the slower growth rates (in 170 mM 2-propanol), and cell viabilities at mid-log phase were not enhanced. Unsurprisingly, no growth was observed under these conditions when using 2-propanol as a sole carbon source (mole ratio of 1.0).

### 3.2. Oxidative extremotolerance under ultraminimal conditions

The extremotolerance of *A. radioresistens* 50v1 toward aqueous hydrogen peroxide (H_2_O_2_) was measured in both nutrient-rich (LB) and ultraminimal (0.2 × M9, 16 mM ethanol, 26 μM Fe^2+^) media. For mid-log phase cultures (1.5 × 10^8^ ± 0.4 × 10^8^ cfu/mL), exposures of 10 mM H_2_O_2_ (for 1 h) in ultraminimal medium resulted in a ∼1.5-log reduction in survival (5.3 × 10^6^ ± 2.4 × 10^6^ cfu/mL). In sharp contrast, exposures to mid-log phase cultures in nutrient-rich medium resulted in no loss in survival (1.9 × 10^9^ ± 0.4 × 10^9^ cfu/mL, 10 mM H_2_O_2_, 1 h). Furthermore, at 100 mM H_2_O_2_, no viability was observed in ultraminimal medium; whereas only a ∼1-log reduction was measured in nutrient-rich medium (3.0 × 10^8^ ± 0.4 × 10^8^ cfu/mL). These results revealed a positive correlation between nutrient availability and survivability, as (expectedly) oxidative extremotolerance was significantly enhanced under nutrient-rich conditions. However, exposures to 10 mM H_2_O_2_ in ultraminimal medium resulted in appreciable survivals, as exposures to ∼5 × 10^6^ cfu/mL yielded only an ∼30-fold loss in viability.

### 3.3. Stable isotope labeling

Cultivation on ethanol as a sole carbon source was confirmed by untargeted metabolomics analysis and stable isotope profiling using ^13^C-labeled ethanol ([1,2-^13^C_2_]-ethanol). Cultures of *A. radioresistens* 50v1 were prepared in ultraminimal medium (0.2 × M9, 26 μM Fe^2+^) using 16 mM ethanol or ^13^C-labeled ethanol, and the cell extracts were analyzed by GC-MS (Supplementary Fig. S2A, B).

As listed in Supplementary Table S1, cultivations on ethanol in ultraminimal medium yielded an array of metabolites, including citric acid cycle intermediates (malate and citrate), mono and disaccharides (fructose, glucose, and trehalose), amino acids (asp, cys, glu, gln, gly, ile, lys, met, phe, pro, ser, thr, trp, tyr, and val), modified amino acids (2-oxoproline, homoserine, ornithine, and β-alanine), peptides (glycylglycine), short and long chain fatty acids (11-octadecenoic acid, 2-butenedioic acid, 2-hexenedioic acid, 2-propenoic acid, 9-octadecenoic acid, butanedioic acid, hydroxybutyric acid, myristic acid, nonanoic acid, palmitic acid, pentanedioic acid, propanoic acid, and stearic acid), fatty alcohols (1-hexadecanol, 1-octadecanol, 2-dodecanol), nucleobases (adenine and pyrimidine), and a variety of other metabolites (*e.g.*, 3-amino-2-piperidone, 3-hydroxyisovaleric acid, 4-hydroxybenzoic acid, 4-hydroxyphenyllactic acid, benzenepropanoic acid, dimethyl tartarate, homogentisic acid, indole-2-carboxylic acid, *N*-ethyldiethanolamine, and oxalic acid).

**FIG. 3.**
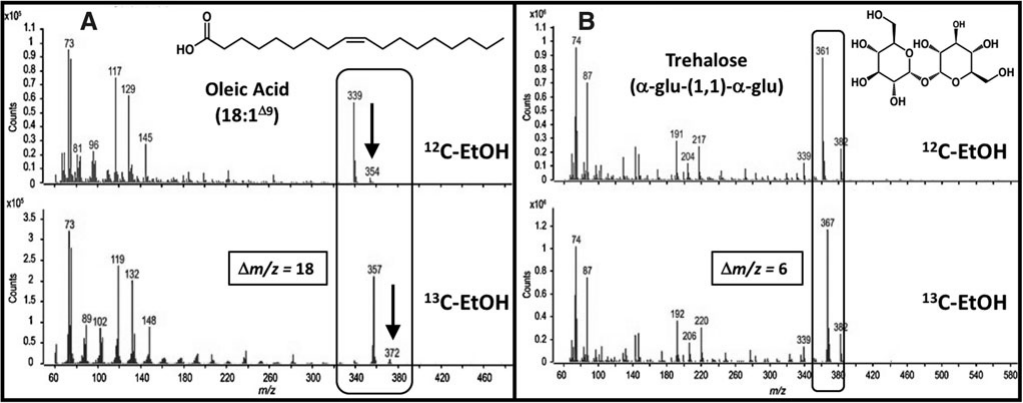
Mass spectra for **(A)** oleic acid (18:1^Δ9^) and **(B)** trehalose [α-d-glucopyranosyl-(1 ↔ 1)-α-d-glucopyranose] extracted from cultures of *A. radioresistens* 50v1 cultivated (32°C) in 0.2 × M9 and 26 μM Fe^2+^ containing 16 mM [1,2-^13^C_2_] ethanol; inset structures represent the underivatized compound, rounded corner boxes highlight the molecular ion (arrow) or fragment ion, and the representative total ion chromatogram is provided in Supplementary Figures S2A and S2B.

Confirmation of ethanol incorporation was obtained by comparison of these metabolites with those extracted from cells cultivated on ^13^C-labeled ethanol. As representative examples, mass spectra for oleic acid [(*E*)-9-octadecenoic acid] and trehalose (α-d-glucopyranosyl-(1 ↔ 1)-α-d-glucopyranose) are provided in Figure 3. In Figure 3A (highlighted by the arrow), the molecular ion for the MSTFA-derivatized version of oleic acid (trimethylsilyl ester of oleic acid, 354.6 g/mol) is observed at a mass-to-charge ratio (*m/z*) of 354. In comparison, mass spectra for oleic acid obtained from cultures grown on ^13^C-labeled ethanol yielded a molecular ion at 372 *m/z*, representing a gain of 18 mass units, consistent with ^13^C incorporation at each of the 18 carbons of oleic acid (18:1^Δ9^). For trehalose, in Figure 3B, the fragment ion at 361 *m/z* represented a six-carbon product resulting from scission at the glycosidic bond to yield two identical fragment ions (Füzfai *et al.*, 2008). In comparison, mass spectra of trehalose obtained from cultures grown on ^13^C-labeled ethanol yielded a fragment ion peak at 367 *m/z*, representing a gain of 6 mass units, consistent with ^13^C incorporation at each of the carbons in the fragment ions.

### 3.4. Kinetics of ethanol and 2-propanol oxidation

**FIG. 4.**
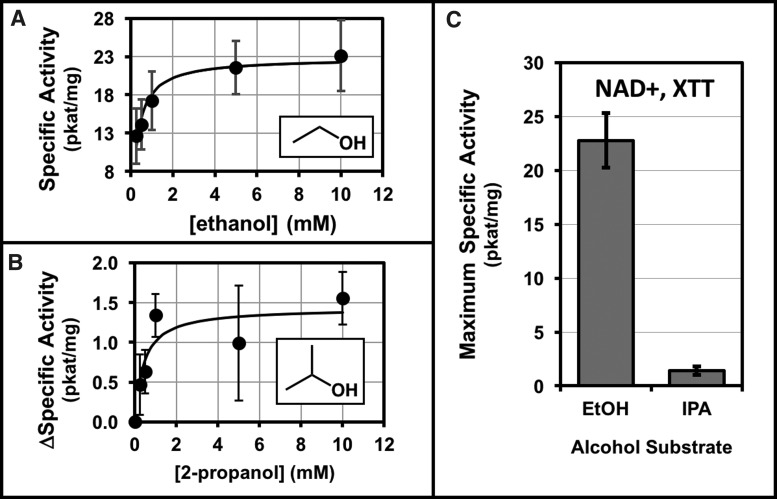
Michaelis–Menten kinetics and nonlinear least-squares regressions (fitted line) for **(A)** ethanol and **(B)** 2-propanol catalysis using suspended membrane fractions of *A. radioresistens* 50v1 (cultivated in 0.2 × M9, 26 μM Fe^2+^, and 16 mM ethanol at 32°C), and **(C)** comparisons of the maximum specific activities (pkat/mg) for ethanol and 2-propanol (error bars represent the standard deviation, *n* = 7–9 for ethanol and *n* = 3–7 for 2-propanol).

The alcohol dehydrogenase activities in cellular extracts of *A. radioresistens* 50v1 were measured against the substrates of ethanol and 2-propanol (Fig. 4). Cultures were prepared under ultraminimal conditions (0.2 × M9, 26 μM Fe^2+^) using 16 mM ethanol as the sole carbon source. Extracts were prepared by ultrasonication, and the membrane (resuspended pellet) and soluble (supernatant) fractions were separately tested for alcohol oxidation activities. Comparisons of the protein extracts revealed approximately fivefold higher specific activities in the membrane fractions. Kinetic studies on the membrane fractions using the substrates of ethanol or 2-propanol (Fig. 4A, B) revealed standard Michaelis–Menten-type behavior, with nonlinear regressions providing maximum specific activities of 23 ± 3 pkat/mg and 1.4 ± 0.4 pkat/mg (and apparent *K*_M_ values of ∼0.3 and ∼0.4 mM), respectively. As shown in Figure 4C, the calculated maximum specific activities for ethanol were ∼16-fold higher than that of 2-propanol.

### 3.5. Biodegradation of Kleenol 30

The biodegradation of Kleenol 30 was measured in cultures of *A. radioresistens* 50v1 prepared in ultraminimal medium (0.2 × M9, 26 μM Fe^2+^) containing 16 mM ethanol with 0.1% or 1.0% v/v Kleenol 30. Growth rates in the presence of 0.1% v/v Kleenol 30 were not impacted (0.46 ± 0.03 h^−1^) when compared with parallel cultures grown in absence of Kleenol 30 (0.48 ± 0.02 h^−1^); however, growth rates decreased ∼1.2-fold in 1.0% v/v Kleenol 30 (0.41 ± 0.02 h^−1^). For all cultures, clarified media (or the extracellular fraction) were prepared by centrifugation and analyzed by GC-MS, and relevant controls included cultures of the (1) 50v1 strain containing no Kleenol 30 and (2) Kleenol 30 incubated for equivalent times in 0.2 × M9 containing 26 μM Fe^2+^, 16 mM ethanol, and no bacteria (Supplementary Fig. S2D, E).

**FIG. 5.**
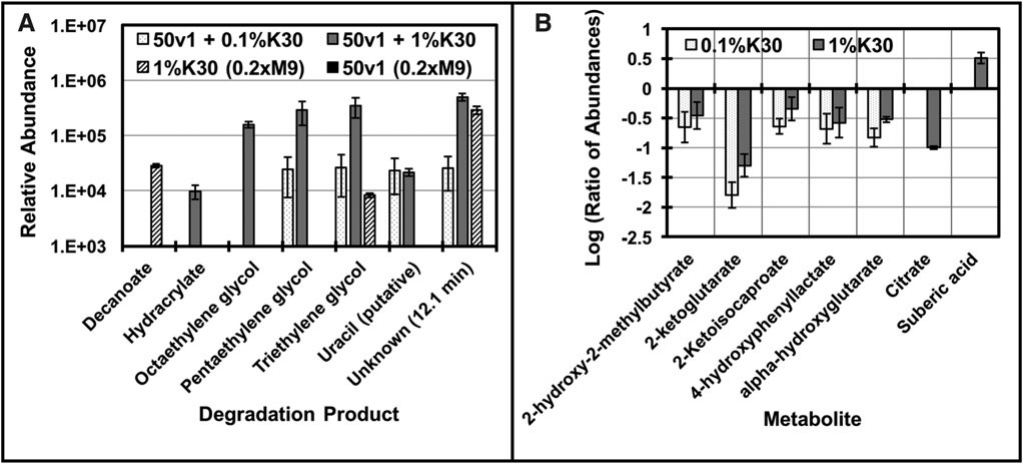
Biodegradation and impacts of 0.1% and 1.0% v/v Kleenol 30 on cultures of *A. radioresistens* 50v1 (0.2 × M9, 26 μM Fe^2+^, 16 mM ethanol, 32°C), where **(A)** the relative abundances of the degradation products for Kleenol 30 (K30) are compared with those in the control samples of (1) K30 incubated in 0.2 × M9 (1%K30) and (2) the 50v1 strain grown in the absence of K30 (50v1 0.2 × M9); **(B)** impacts on the extracellular metabolites, as displayed by the logarithm of the ratio of abundances measured in the presence of K30 (0.1% and 1.0%) and absence of K30 (50v1 0.2 × M9, control sample), where negative values indicated a decrease in abundance compared with the control, and positive values indicated an increase in abundance compared with the control (for these experiments, biological replicates referred to samples cultivated from plates prepared from glycerol stocks, and technical replicates referred to samples cultivated from separate colonies from the same plate; all metabolites were detected in three biological replicates [*n* = 3], with at least one technical replicate per condition, except for citrate [*n* = 1 with three technical replicates] and suberic acid [*n* = 1 with two technical replicates]; error bars represent **(A)** the standard deviation and **(B)** propagated error).

Comparison of the data (*p* < 0.0171, *t*-test, false discovery rate of 0.10) supported the formation of degradation products, impacts to the extracellular metabolome, and potential metabolism of a component of Kleenol 30 (Fig. 5). As summarized in Figure 5A, compounds (retention time [RT], min) including hydracrylate (RT 8.2), octaethylene glycol (RT 25.0), pentaethylene glycol (RT 18.6), triethylene glycol (RT 16.0), and uracil (putative assignment, RT 10.8) were only detected in the presence of Kleenol 30 and *A. radioresistens* 50v1. In comparison, these compounds were undetectable (or below the limit of detection) in the control samples. Together, this directly supported biodegradation of Kleenol 30 (presumably a polymeric ethylene glycol formulation) into lower molecular weight and volatilizable constituents. In context, the known components of Kleenol 30 (http://hazard.com/msds/f2/byw/bywhr.html) include 12.5% ethylene glycol monobutyl ether, 1–5% nonylphenol ethoxylate, 1% dodecylbenzenesulfonate, and 1–4% silicic acid, disodium salt.

As summarized in Figure 5B, impacts to the extracellular metabolome were supported by decreases in abundances for several hydroxy- and ketoacids, including 2-ketoglutarate, α-hydroxyglutarate, 2-ketoisocaproate, citrate, and 4-hydroxyphenyllactate. Furthermore, control experiments with Kleenol 30 (Fig. 5A) showed that incubations in 0.2 × M9 (containing 26 μM Fe^2+^ and 16 mM ethanol), in the absence of *A. radioresistens* 50v1, yielded substantial increases in the abundances of decanoate (10:0). However, in the presence of *A. radioresistens* 50v1, decanoate was not detected, suggesting either biodegradation or metabolism of this product.

## 4. Discussion

In this study, we measured the ability of several strains of spacecraft-associated *Acinetobacter* to metabolize or biodegrade the reagents used to clean spacecraft, and surfaces and floors of spacecraft assembly facilities. Cultivations in the presence of the cleaning reagents were conducted under ultraminimal conditions to roughly approximate the low-nutrient and low-particulate (inorganics and organics) conditions of the assembly facilities. Cultivations were performed in 0.2 × M9 minimal medium supplemented with 26 μM Fe^2+^, where alkali, halogen, and main group elements (*i.e.*, N, S, and P, excluding oxygen) were <15 mM in concentration, alkaline earth metals were ≤0.4 μM, and heavy metals contaminants were collectively ≤0.001% of the medium constituents. In comparison with prior work, early cultivations of the *Acinetobacter* on ethanol relied upon inorganic-rich medium (Abbott *et al.*, 1973) containing a variety of supplemented metalloids and transition metals (*e.g.*, 0.1–100 μM concentrations of Fe, Mn, Cu, Co, Zn, Mo, and B), with other media containing organic components such as citrate (Du Preez *et al.*, 1981), and yeast autolysate and pantothenate (Pirog and Kuz'minskaya, 2003).

Under more extreme nutrient-restricted conditions (0.2 × M9, 26 μM Fe^2+^), most of the tested spacecraft-associated strains grew on ethanol as a sole carbon source (six out of the seven tested strains). Cultivations provided high cell counts at mid-log phase (10^8^–10^9^ cfu/mL), where generation times (or doubling times) ranged from 0.56 to 0.80 h in 16 mM (0.1% v/v) ethanol, and from 0.85 to 1.1 h in 160 mM (1.0% v/v) ethanol. All viable spacecraft-associated strains grew optimally at lower ethanol concentrations, with *A. radioresistens* 50v1 having an optimal substrate concentration range with an upper limit of 2–40 mM. In contrast, the *A. radioresistens* type strain did not exhibit an optimal substrate concentration, and generation times (at ≤16 mM ethanol) were approximately twofold slower than all tested strains. These comparisons support a trend between lower optimal substrate concentrations and spacecraft association, which would be a necessary correlation for any postulated metabolism under the oligotrophic conditions of the assembly facilities.

For this study, all downstream analyses focused on *A. radioresistens* 50v1, which is the best characterized strain among the spacecraft-associated *Acinetobacter*; in addition, at the species level, *A. radioresistens* has been detected on Mars-bound spacecraft and in the ISS (surfaces and drinking water) (La Duc *et al.*, 2003; Castro *et al.*, 2004; McCoy *et al.*, 2012; Moissl-Eichinger *et al.*, 2013; Schuerger *et al.*, 2013; Derecho *et al.*, 2014). For *A. radioresistens* 50v1, use of ethanol as a sole carbon source was confirmed through stable isotope labeling (using ^13^C-labeled ethanol) and untargeted analyses, which supported full enrichment of ^13^C in the primary metabolome. Consistent with the known microbiology of *A. radioresistens* (Nishimura *et al.*, 1988), 2-propanol (isopropyl alcohol, isopropanol) did not serve as a sole carbon source; however, cultivations on mixtures of ethanol and 2-propanol (70/30 and 50/50 mixtures) showed enhanced growth, as indicated by statistically significant increases in plate counts (cfu/mL) at mole ratios of ≤0.50.

Biochemical interrogations supported the oxidation of both ethanol and 2-propanol by extracts of *A. radioresistens* 50v1, likely due to a membrane-associated alcohol dehydrogenase (Singer and Finnerty, 1985). Michaelis–Menten kinetics provided a low *K*_M_ value for ethanol (∼0.3 μM), consistent with the faster generation times obtained at the lower ethanol concentrations in ultraminimal medium. Kinetic studies also indicated a requisite need for both NAD^+^ and an exogenous electron acceptor (XTT or DCIP), thereby supporting the formation of NADH (although transiently in cellular extracts) from either alcohol substrate, which *in vivo* (and in the presence of intact membranes) would directly support respiration.

Molecular and cultivation studies show that the floor of the assembly facilities also harbor strains of *Acinetobacter* (Ghosh *et al.*, 2010; La Duc *et al.*, 2012), with human-based activities possibly assisting in physical transport of these microbes across the facilities. Molecular experiments with *A. radioresistens* 50v1 show that Kleenol 30, an alkaline floor detergent, is biodegraded under ultraminimal conditions into lower molecular weight ethylene glycols.

Furthermore, untargeted analysis of the extracellular metabolome showed decreased abundances for several hydroxy- and ketoacids in the presence of Kleenol 30. Interestingly, these hydroxy- and ketoacids share iron-binding properties (Drechsel *et al.*, 1993; Schofield and Zhang, 1999; Yue *et al.*, 2003) and, hence, reveal a potential impact to the transport/metabolism of extracellular iron. Our studies also indicate that decanoate (formed during incubation in Kleenol 30 in 0.2 × M9/Fe/ethanol) is biodegraded by *A. radioresistens* 50v1 (Fig. 5), with the results (Fig. 5B) suggesting concomitant increases in abundances of suberic acid (octanedioic acid), which along with acetyl-CoA would potentially be a metabolic product of ω-oxidation of decanoate (Donoghue and Trudgill, 1975; Kunz and Weimer, 1983; Van Bogaert *et al.*, 2011).

Under ultraminimal conditions, we also show that *A. radioresistens* 50v1 exhibits a remarkable oxidative extremotolerance when cultivated on ethanol as a sole carbon source (∼1.5-log reduction, ∼10^8^ cfu/mL, 10 mM H_2_O_2_). This assessment is based on comparisons with other nonspore forming radiation and oxidation-resistant bacteria. For instance, the survivability of *A. radioresistens* 50v1 in nutrient-poor medium is comparable with that of *Deinococcus radiodurans* R1 (∼1-log reduction in 33 mM H_2_O_2_) and that of *Vibrio rumoiensis* S-1T (∼1.5-log reduction in 0.4 mM H_2_O_2_) when cultivated in nutrient-rich medium (*e.g.*, LB, tryptic soy broth, and peptone yeast extract starch) (Arrage *et al.*, 1993; Ichise *et al.*, 1999). Hence, by extension, these results support the potential for oxidative extremotolerance under oligotrophic conditions, which is significant, as desiccating environments (such as the assembly facilities) are thought to promote oxidative stress through the formation of reactive oxygen species (Billi and Potts, 2002; Franca *et al.*, 2007).

In the context of survival in the assembly facilities, therefore, these combined results support the potential for ethanol, 2-propanol, and perhaps Kleenol 30 to (1) serve as carbon or energy sources under oligotrophic conditions and (2) sustain extremotolerances against the oxidative stresses associated with low-humidity environments. For recent Mars missions, however, the surface cleaning procedures were predominantly performed using isopropyl alcohol wipes (rather than ethanol) (La Duc *et al.*, 2012; Benardini *et al.*, 2014). Accordingly, and as a potential survival mechanism, members of the core microbiome likely remained on the surface after wiping, wetting by the residual cleaning reagents likely initiated a basal metabolic activity, and the resulting activity was likely attenuated upon evaporation of the cleaning reagents (analogous to our experiments using loosely capped culture tubes).

In turn, the cycles of wetting/drying, resulting from high-frequency cleaning, likely imposed certain stresses (such as oxidative stress) on the microorganisms, with the residual evaporates forming a baseline vapor abundance in the facilities. In support, recent measurements show that the vapor abundances of 2-propanol are ∼0.1 ppm in facilities maintained at ISO classes 8 and 7 standards (Dworkin *et al.*, 2018); hence, these observations support the potential for 2-propanol to serve as a perpetual nutrient source, as the acquisition of volatile organics is a known survival tactic for soil bacteria (Hanzel *et al.*, 2011; Modrzyński *et al.*, 2016). In this combined perspective, and after substrate acquisition, the *Acinetobacter* could have slowly metabolized 2-propanol to yield minimal but potentially sufficient amounts of NADH to support survival. Moreover, when accounting for surface communities within the assembly facilities, our work opens the possibility that metabolic contributions may arise from other members of the core spacecraft microbiome.

## 5. Conclusion

In sum, this study provides a plausible biochemical rationale to the observed microbial ecology dynamics of spacecraft assembly facilities, as spacecraft-associated microorganisms (such as the *Acinetobacter*) may metabolize/biodegrade spacecraft cleaning reagents and exhibit extreme oxidative tolerances under the oligotrophic and low-humidity conditions. This work also adds to the range of known survival features for the spacecraft-associated *Acinetobacter*, which include extreme tolerances toward aqueous hydrogen peroxide, under nutrient-poor (*this study*) and nutrient-rich conditions (Derecho *et al.*, 2014), desiccation (McCoy *et al.*, 2012), sequential exposures to oxidative and radiative stressors (McCoy *et al.*, 2012), heat treatments (80°C for 15 min) (Moissl-Eichinger *et al.*, 2013), and exposures to martian atmospheric and pressure conditions (Schuerger *et al.*, 2013).

In the framework of planetary protection, therefore, the Gram-negative and nonspore forming *Acinetobacter* may tolerate partial sterilizations with vaporous hydrogen peroxide, and survive the heat treatments associated with the NASA Standard Assay, which could possibly impact treatments and measurements for missions requiring very low bioburden values (*e.g.*, life detection and Special Regions missions). Furthermore, our work lends support toward the use of differing (and rotating) spacecraft-compatible cleaning reagents as a means of controlling the core spacecraft microbiome.

## Supplementary Material

Supplemental data

Supplemental data

Supplemental data

## Notes

Associate Editor: Petra Rettberg

## Abbreviations Used

FAMEs: fatty acid methyl esters
GC-MS: gas chromatography–mass spectrometry
ISS: International Space Station
LB: Lysogeny broth
MSTFA: *N*-methyl-*N*-(trimethylsilyl)trifluoroacetamide
OD: optical density
PBS: phosphate-buffered saline
RT: retention time
