# Supplementary material for: Metabolism and Biodegradation of Spacecraft Cleaning Reagents by Strains of Spacecraft-Associated Acinetobacter
Source: Astrobiology. 2018 Nov 29;18(12):1517–27. doi: 10.1089/ast.2017.1814 (PMC6276816; doi:10.1089/ast.2017.1814)
Supplement: Supplemental data [file Supp_Fig1.pdf]

Supplementary Data

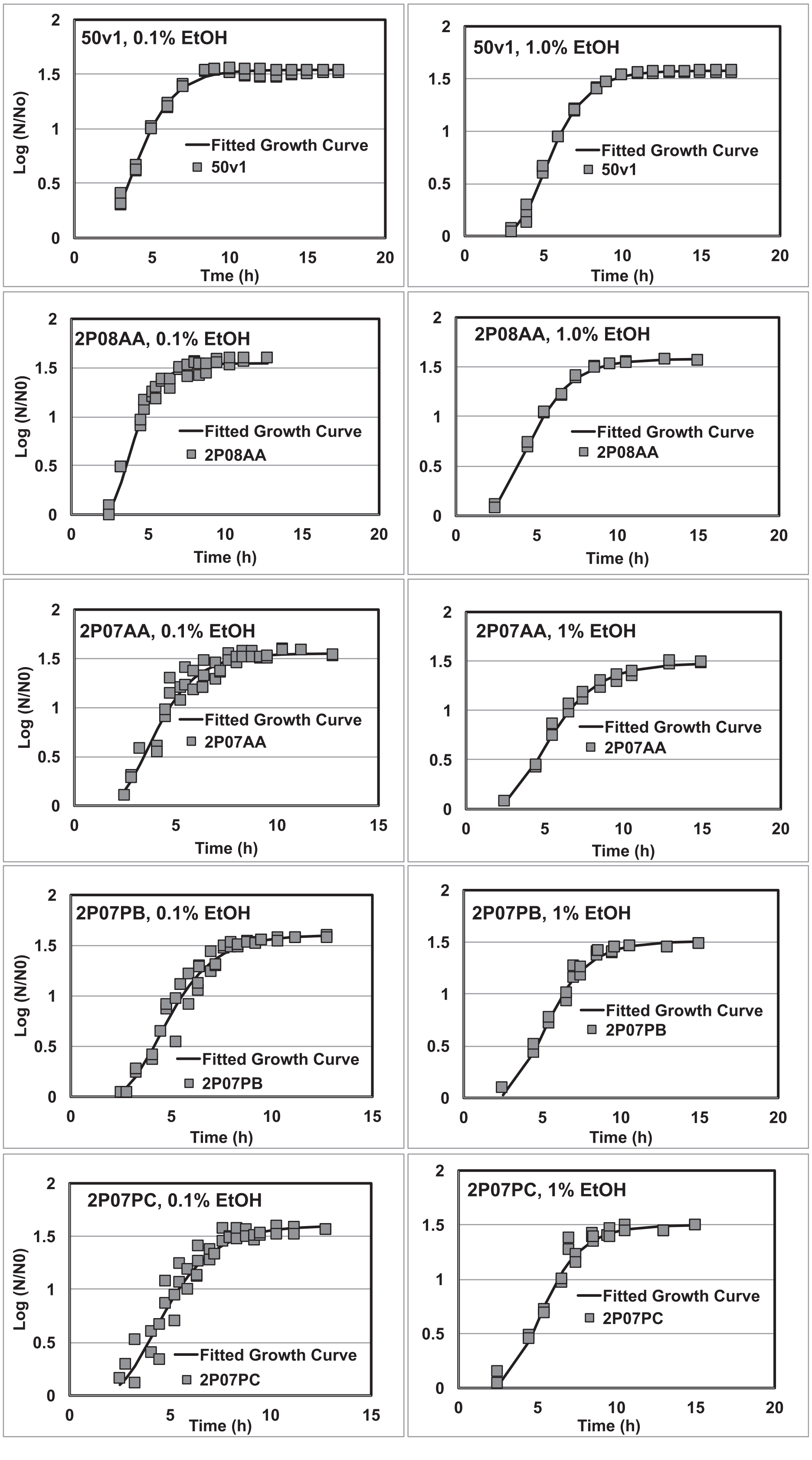

SUPPLEMENTARY FIG. S1. Growth curves and regression analyses for *Acinetobacter radioresistens* 50v1, *Acinetobacter proteolyticus* 2P01AA, *Acinetobacter johnsonii* 2P08AA, *A. johnsonii* 2P07AA, *Acinetobacter oryzare* 2P08MC, *Acinetobacter guillouiae* 2P07PB, and *A. guillouiae* 2P07PC; cultivations were performed at 32°C in 0.2×M9 containing 26 μM Fe^{2+} and 16 mM (0.1% v/v) or 160 mM (1.0% v/v) ethanol; regressions were performed using Equation 1, and data points from multiple biological replicates are displayed (n = 3–4).
