# Supplementary material for: Metabolism and Biodegradation of Spacecraft Cleaning Reagents by Strains of Spacecraft-Associated Acinetobacter
Source: Astrobiology. 2018 Nov 29;18(12):1517–27. doi: 10.1089/ast.2017.1814 (PMC6276816; doi:10.1089/ast.2017.1814)
Supplement: Supplemental data [file Supp_Fig2.pdf]

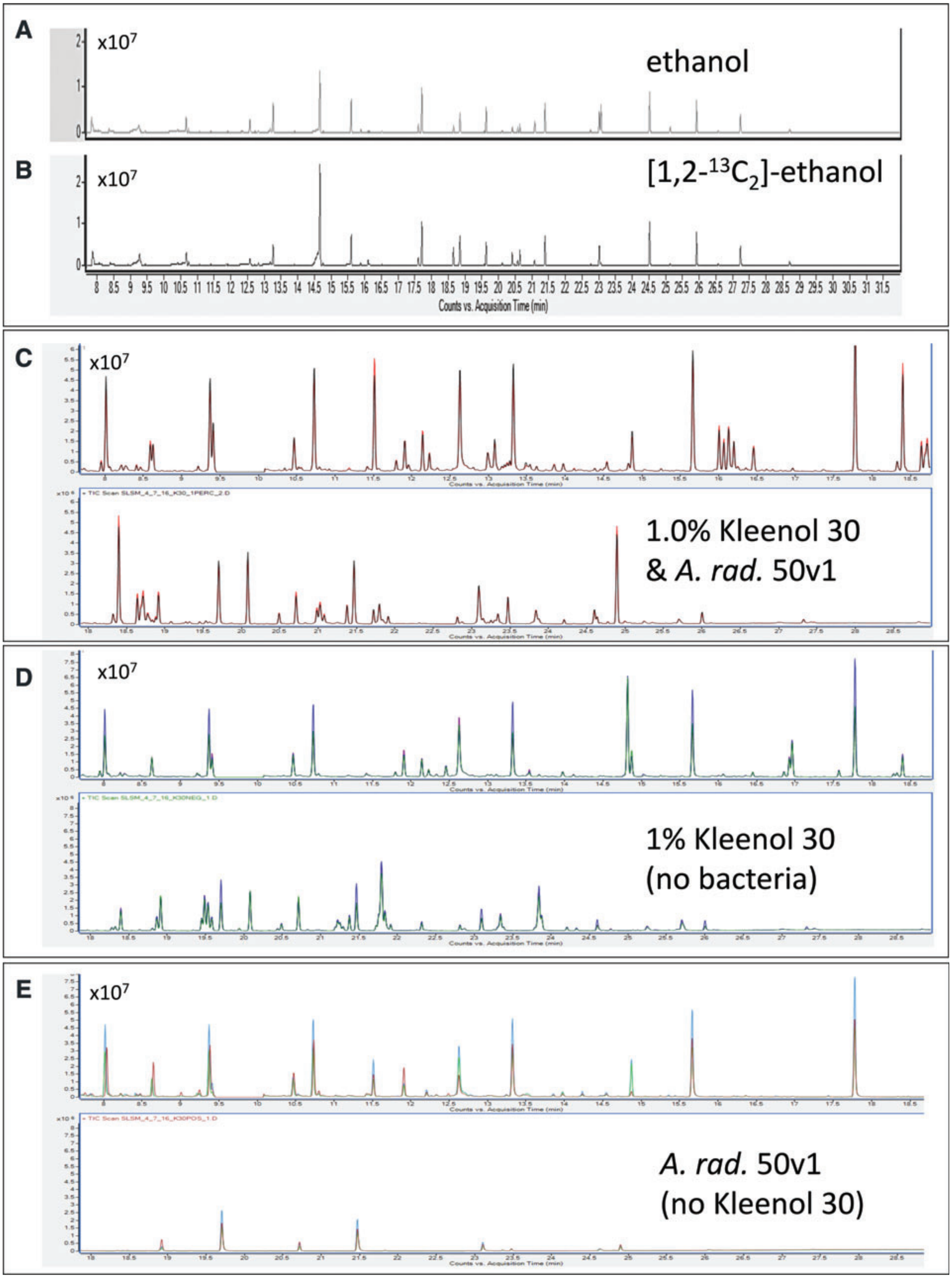


**SUPPLEMENTARY FIG. S2.** Representative total ion chromatograms from GC-MS analysis of extracts of *A. radioresistens* 50v1 cultivated (32°C) in 0.2×M9 and 26 $\mu$M $Fe^{2+}$ containing **(A)** 16 mM ethanol, **(B)** [1,2-$^{13}C_2$]-ethanol, and **(C)** 1.0% v/v Kleenol 30, including the respective controls of **(D)** Kleenol 30 incubated in 0.2×M9, and **(E)** the 50v1 strain grown in the absence of Kleenol 30; for **(C–E)** the upper chromatograms show retention times 8.5–18.5 min, whereas the lower chromatograms show retention times 18–28.5 min. GC-MS, gas chromatography–mass spectrometry.
