# Supplementary material for: Metabolism and Biodegradation of Spacecraft Cleaning Reagents by Strains of Spacecraft-Associated Acinetobacter
Source: Astrobiology. 2018 Nov 29;18(12):1517–27. doi: 10.1089/ast.2017.1814 (PMC6276816; doi:10.1089/ast.2017.1814)
Supplement: Supplemental data [file Supp_Table1.pdf]

Metabolite & Retention Time (min)

Citric Acid Cycle

Citric acid	16.6
Malic acid	12.8

Mono and Disaccharides

Fructose	17.1
Glucose	17.4
Trehalose	24.6

Amino Acids

Aspartic acid	13.2
Cysteine	13.6
Glutamic acid	14.4
Glutamine	16.1
Glycine	10.4
Isoleucine	10.2
Lysine 3TMS	15.4
Lysine 4TMS	17.7
Methionine	13.2
Phenylalanine	14.5
Proline	10.3
Serine	11.1
Threonine	11.5
Tryptophan	20.5
Tyrosine	17.8
Valine	9.2

Modified Amino Acids

5-Oxoproline	13.2
Homoserine	12.3
Ornithine	14.3
β-Alanine	12.0

Peptide

Glycylglycine	16.2

Short and Long Chain Acids

11-Octadecenoic acid	20.6
2-Butenedioic acid	11.0
2-Hexenedioic acid	12.9
2-Propenoic acid	14.1
(*E*)-9-Octadecenoic acid	19.4
Butanedioic acid	10.5
Hydroxybutyric acid	8.3
Myristic acid	17.0
Nonanoic acid	11.2
Palmitic acid	18.9
Pentanedioic acid	13.8
Propanoic acid	16.4
Stearic acid	20.7

Fatty Alcohols

1-Hexadecanol	18.1
1-Octadecanol	20.0
2-Dodecanol	18.1

Nucleobases

Adenine	17.2
Pyrimidine	11.7

Other Metabolites

3-Amino-2-piperidone	12.4
3-Hydroxyisovaleric acid	9.1
4-Hydroxybenzoic acid	14.5
4-Hydroxyphenyllactic acid	17.4
Benzenepropanoic acid	14.1
Dimethyl tartarate	8.2
Homogentisic acid	16.4
Indole-2-carboxylic acid	20.9
N-Ethyldiethanolamine	11.5
Oxalic acid	8.2
